# Identification and characterization of RASSF1C piRNA target genes in lung cancer cells

**DOI:** 10.18632/oncotarget.15965

**Published:** 2017-03-07

**Authors:** Mark E Reeves, Mathew Firek, Abdullaati Jliedi, Yousef G Amaar

**Affiliations:** ^1^ Surgical Oncology Laboratory, Loma Linda VA Medical Center, Loma Linda, California, USA; ^2^ Loma Linda University Cancer Center, Loma Linda, California, USA

**Keywords:** lung cancer, RASSF1C, PIWIL1, piRNAs, cell proliferation

## Abstract

RASSF1C up-regulates important genes involved in lung cancer cell growth, including a stem cell self-renewal gene, *piwil1*. In this article, we report the identification of small noncoding PIWI-interacting RNAs (piRNAs) in lung cancer cells over-expressing RASSF1C. A piRNA microarray screen was performed using RNA isolated from the lung cancer cell line H1299 stably over-expressing RASSF1C and corresponding control. The piRNA microarray screen identified several piRNAs that are regulated by RASSF1C and we have validated the expression of two up-regulated piRNAs (piR-34871 and piR-52200) and two down-regulated piRNAs (piR-35127 and piR-46545) in lung cancer cells with silenced and over-expressed RASSF1C using RT-PCR. We also assessed the expression of these four piRNAs in lung tumor and matched normal tissues (*n* = 12). We found that piR-34871 and piR-52200 were up-regulated in 58% and 50%, respectively; while piR-35127 and piR-46545 were down-regulated in 50% in lung tumor tissues tested. The expression of piR-35127 was inversely correlated with RASSF1C expression in 10/12 tumor tissues. Over-expression of piR-35127 and piR-46545 and knock-down of piR-34871 and piR-52200 significantly reduced normal lung and breast epithelial cell proliferation and cell colony formation as well as proliferation of lung cancer cell lines (A549 and H1299) and breast cancer cell lines (Hs578T and MDA-MB-231). This suggests that these novel piRNAs may potentially be involved in regulating lung cell transformation and tumorigenesis. RASSF1C may potentially modulate the expression of its piRNA target genes through attenuation of the AMPK pathway, as over-expression of RASSF1C resulted in reduction of p-AMPK, p21, and p27 protein levels.

## INTRODUCTION

Lung cancer kills more people than any other cancer in the United States [1–2]. The RASSF1 gene encodes two major isoforms, RASSF1A and RASSF1C, derived by alternative promoter selection and mRNA splicing [3–5]. RASSF1A is a tumor suppressor, whereas RASSF1C is emerging as a cancer cell growth and migration promoter [4–18]. We have discovered that RASSF1C regulates a stem cell renewal gene, *piwil1*, in lung cancer cells, suggesting a potential role for RASSF1C in lung cancer stem cell (CSC) growth and progression [14, 17]. We have also demonstrated that RASSF1C overexpression induces phosphorylation of ERK1/2 in lung cancer cells. This suggests the hypothesis that RASSF1C exerts its actions on target genes such as *piwil1*, in part, through the activation of the MAP-ERK1/2 pathway [14]. PIWI-like proteins are a subfamily of Argonaut proteins that interact with small PIWI-interacting RNA molecules (piRNAs) which are small RNAs 24–32 nucleotides in length to form complexes that regulate transcriptional and translational repression leading to inhibition of apoptosis, stimulation of cell division and proliferation, and down-regulation of cyclin inhibitors and tumor suppressors [19, 20, 21]. Indeed, higher PIWIL1 expression has been reported in several tumor types (testicular, breast, endometrial, gastrointestinal, ovarian, prostate, soft-tissue sarcoma and seminomas) compared to the corresponding normal tissues [22–26]. Modulation of PIWIL1 and piRNA gene expression by RASSF1C is potentially a novel and important mechanism that may contribute to lung cancer stem cell development and progression. To further investigate our hypothesis and to learn more about the underlying mechanism(s), we carried out a global piRNA microarray screen to identify piRNAs that are regulated by RASSF1C in non-small cell lung cancer (NSCLC) cells. In this article, we report on the identification of several RASSF1C piRNA target genes in NSCLC cells. We have assessed the expression of specific piRNAs (piR-35127, piR046545, piR-34871, and piR-52200) in lung cancer cells and lung tumor tissues. We have also modulated the expression of these four piRNAs to learn about their function and impact on normal lung and breast epithelial and lung and breast cancer cell proliferation. We also report that RASSF1C may modulate PIWIL1-piRNA gene expression, in part, through inactivation of the AMPK pathway and downstream effectors p21 and p27.

## RESULTS

### PiRNA screen and data analysis

**Figure 1:**
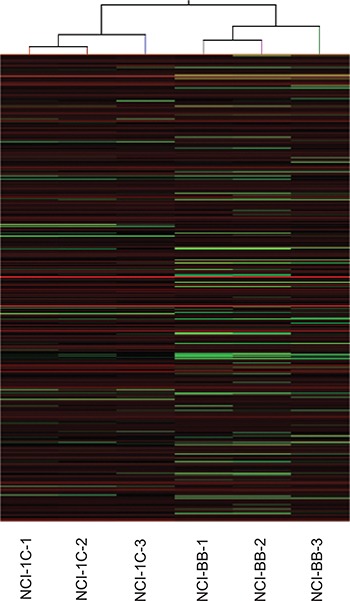
PiRNA expression profiling Hierarchical Clustering for “All Targets Value”. “Red” indicates high relative expression, and “green” indicates low relative expression. The Hierarchical Clustering shows a distinguishable piRNA expression profiling among samples. The lung cancer cell line H1299 stably expressing RASSF1C and the corresponding control (NCI-BB) were used to perform the piRNA microarray in triplicate.

**Figure 2:**
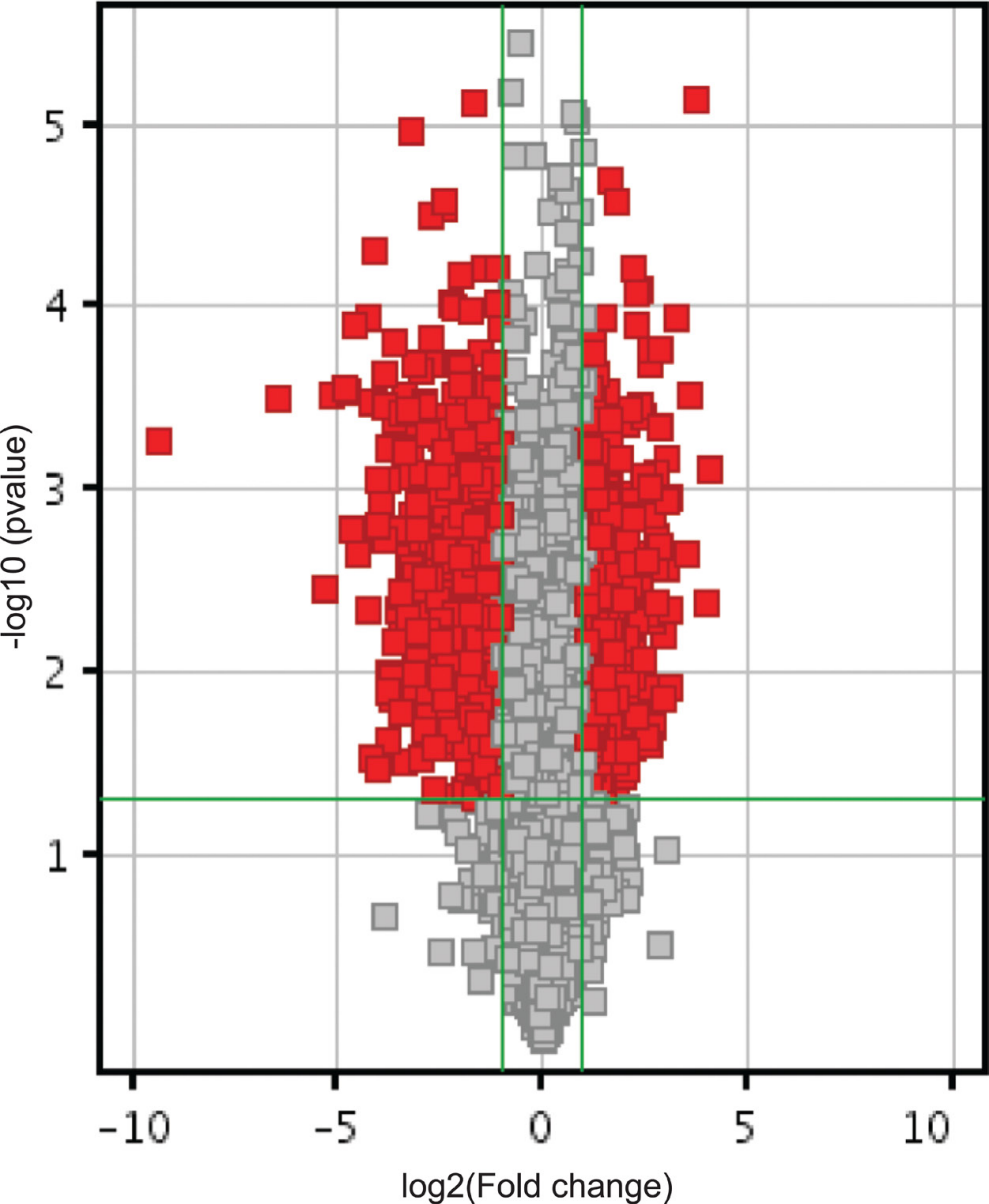
RASSF1C piRNA target gene expression Volcano plot shows piRNA differential expression in lung cancer cells over-expressing RASSF1C and controls using fold-change values and *P*-values. The vertical green lines correspond to 2.0-fold up and down, respectively, and the horizontal green line represents a *P-value* of 0.05. The red points in the plot represent the differentially expressed piRNAs with statistical significance. Over 500 piRNAs that are either up-regulated or down-regulated by RASSF1C are present in the lung cancer cell line H1299.

A global Arraystar piRNA microarray screen profiling 23000 human piRNAs was conducted using RNA isolated from the NCI-H1299 NSCLC cell line over-expressing RASSF1C (NCI-1C, experimental) and corresponding NCI-H1299 over-expressing the vector backbone (NCI-BB, control) cells. The screen and data analysis were performed by Arraystar Inc. A box plot (Figure 1) shows a very similar normalized log2-ratio distribution of intensities of the NCI-1C and NCI-BB sample replicas. To identify differentially expressed piRNAs with statistical significance, a Volcano Plot filtering between the experimental and control groups with a threshold fold change > = 2.0 and *p-value* < = 0.05 was performed (Figure 2). Several hundred piRNAs that appear to up-regulated and down-regulated by RASSF1C have been identified.

### RT-PCR validation of selected RASSF1C-target piRNAs

**Figure 3:**
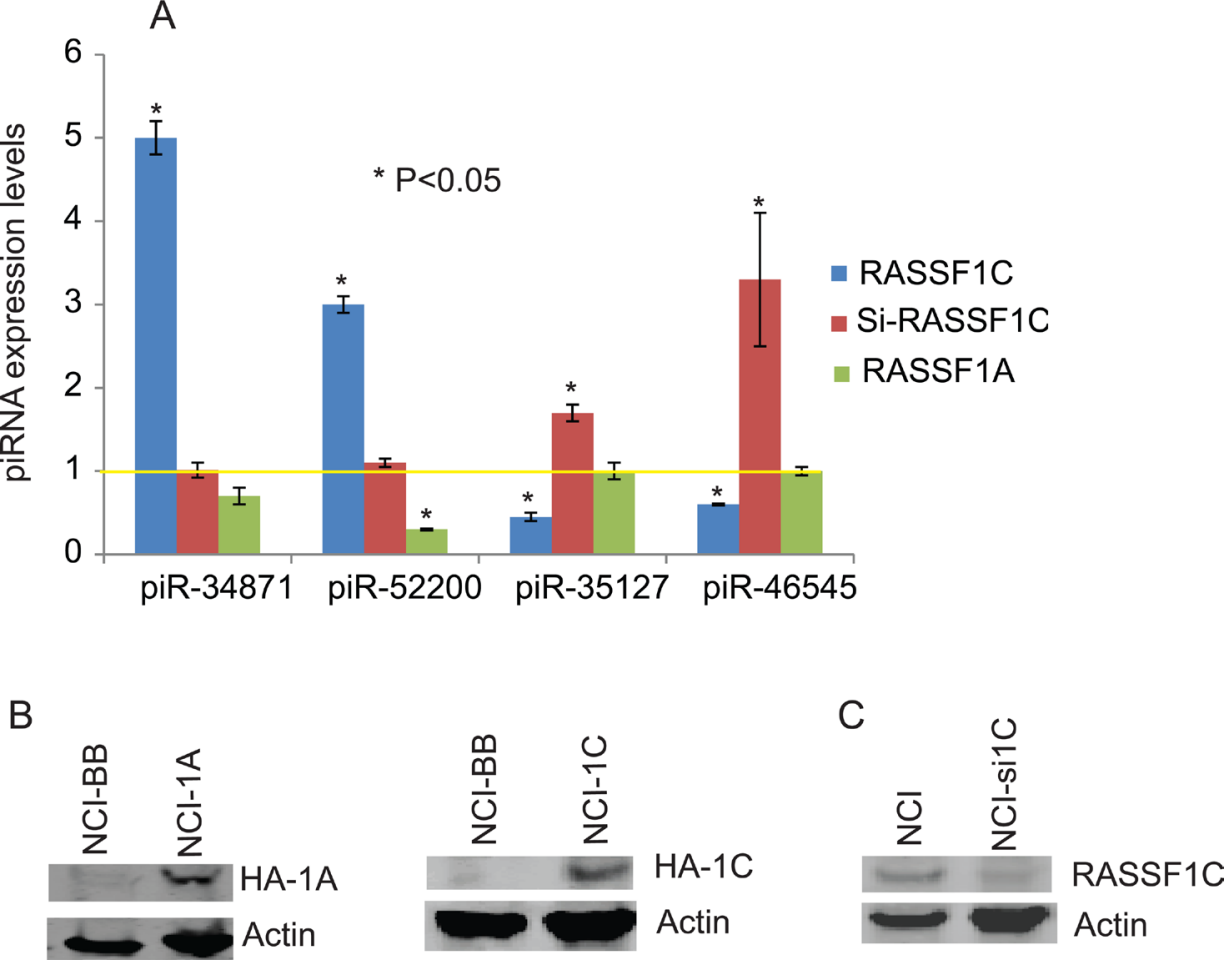
RT-PCR analysis of select piRNAs (**A**) Validation of selected RASSF1C piRNA target genes by RT-PCR in NCI-H1299 lung cancer cells with over-expressed RASSF1C (NCI-1C), silenced RASSF1C (Si-RASSF1C), or over-expressed RASSF1A (NCI-1A) compared to NCI-H1299 –vector backbone (NCI-BB) control cells. The yellow line represents basal piRNA expression in control cells (NCI-BB). The RT-PCR of controls and experimental reactions were run in triplicate in multiple runs and the 2^–ΔΔ^ method was used to perform statistical analysis (32), with a *P <* 0.05. (**B**) Immunoblots showing over-expression of RASSF1A (NCI-1A) and RASSF1C (NCI-1C) in NCI-H1299 cells. HA-tag antibody was used to detect HA-RASSF1 and HA-RASSF1C fusion proteins. (**C**) Immunoblots shows down-regulation of RASSF1C expression (NCI-si1C) in NCI-H1299 cells.

The piRNA screen identified several piRNAs that are regulated by RASSF1C. Selected piRNAs that are up-regulated or down-regulated by RASSF1C in lung cancer cells are listed in Table 1. The expression of four of these piRNAs has been confirmed by RT-PCR analysis in H1299 cells over-expressing RASSF1C or RASSF1A and in H1299 cells with RASSF1C-expression knocked down. The expression of piR-34871 and piR-52200 are up-regulated while piR-35127 and piR-46545 are down-regulated in cells over-expressing RASSF1C (Figure 3). Knocking down RASSF1C by siRNA resulted in increased piR-46545 and piR-35127 expression (Figure 3). In contrast, over-expression of RASSF1A down-regulated the expression of piR-52200 but it did not affect the expression of the expression of piR-34871, piR-35127, and piR-46545 (Figure 3).

**Table 1:** Selected RASSF1C piRNA target genes identified in lung cancer cells using a global piRNA array screen

Up-regulated PiRNAS		Down-Regulated PiRNAs	
PiRNA	Fold Change	PiRNA	Fold Change
piR-34871	9	piR-35127	–16
piR-52200	11	piR-46545	–8
piR-55557	8	piR-31335	–5
piR-33526	7	piR-41263	–3
piR-35527	8	piR-60643	–5
piR-53638	8	piR-33819	–6
piR-39729	7	piR-34393	–4
piR-31143	3	piR-30060	–3
piR-35284	4	piR-33819	–4
piR-36225	3	piR-50485	–3

### Expression of piRNAs in lung tumor tissues

**Figure 4:**
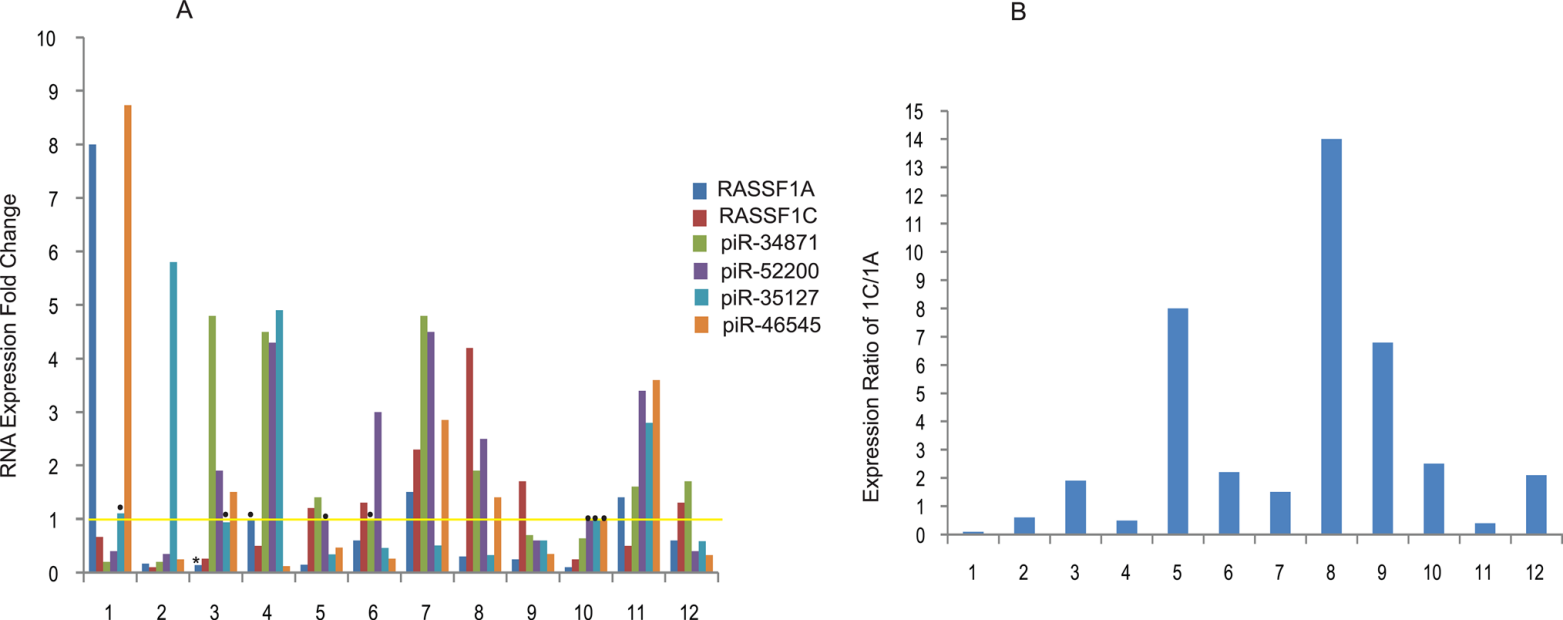
Expression profiling of selected piRNAs in lung tumors (**A**) Expression of RASSF1C, RASSF1A, and selected piRNAs was assessed in lung tumor samples by RT-PCR using gene-specific primers. RASSF1C expression is higher in 6 of 12 tumor samples and RASSF1C expression appears to negatively correlate with that of piR-35127 in 10 of the 12 tumor samples tested. The yellow line represents normalized basal piRNA expression in control samples and thus experimental values above the line represent elevated expression and values below the line represent reduced expression of the piRNA expression. The RT-PCR of control and experimental reactions were run in triplicates in multiple independent runs and the 2^-ΔΔ^method was used to perform statistical analysis (32). The expression fold change (bars) are statistically significant (*P <* .05) compared to controls except those bars with dot (٠) placed on top. (**B**) The ratio of RASSF1C to RASSF1A expression was assessed in 12 lung tumor samples. The ratio of RASSF1C to RASSF1A was > 1 in 8/12 (67%) of lung tumors. The ratio was calculated by dividing [RASSF1C expression in tumor tissue/RASSF1C in normal tissue] by [RASSF1A expression in tumor tissue/RASSF1A in normal tissue].

We have initiated studies to determine the expression of some of the up-regulated and down-regulated piRNAs in lung tumor and matched normal tissues. PiR-34871 and piR-52200 were significantly up-regulated in about 50–58% of tumor tissues (Figure 4), while piR-35127 and piR-46545 were down-regulated in about 50% of tumor tissues. We also compared the level of RASSF1C expression to that of its piRNAs targets and found that there was an expression correlation between RASSF1C and its targets piR-34871, piR-52200, and piR-46545 in some tumor tissues (Figure 4). Six tumor samples (50%) exhibited increased RASSF1C expression and 7 tumor samples (58%) exhibited RASSF1C /RASSF1A ratio > 1. Tumor samples with elevated RASSF1C expression also showed increases in either piR-34871 or piR-52200 expression or both. The expression of piR-35127 showed a distinct inverse correlation with RASSF1C expression in 10/12 (83%) tumor tissues examined. This suggests that piR-35127 may be an authentic and important gene target for RASSF1C. We also assessed the expression of RASSF1A in the same tumor samples and found that RASSF1A expression was down-regulated in 7 of the 12 tumor samples tested. RASSF1A was significantly over-expressed in 3/12 of the tumor tissues examined (Figure 4). Our findings suggest that higher RASSF1C expression and/or higher RASSF1C/RASSF1A ratio appears to impact the modulation RASSF1C target genes.

### Target genes for piRNAs

Sequence alignments were performed using mature sequences of piRNAs as query to run a blast search to identify potential sources and mRNA targets for piR-34871, piR-52200, piR-46545, and piR-35127. Potential sources and piRNA target genes are listed in Table 2. All the piRNAs searched are derived from nuclear genes; piR-34871 appears to also be derived from a mitochondrial gene. The identified mRNA targets for piRNAs that exhibited at least 13 contiguous nucleotide complementary matches are considered potential targets (Table 2).

**Table 2:** Potential sources of piRNAs and piRNA targets were identified using blast sequence alignment for using the mature piRNA sequence as query

PiRNA ID	Potential sources for piRNA	Potential gene targets for piRNA
DQ596805:		
piR-31871	Mitochondrion DNA:	Putative methyltransferase: Seq ID: ref|NC_018914.2|
	Seq ID: ref|NC_012920.1|	Ephrin type-A receptor 6a: Seq ID: ref|NC_018914.2|
	TKIDN2 cDNA clone:	RNA-binding motif, single-stranded
	Seq ID: dbj|DB169298.1	interacting protein 3: Seq ID: ref|NC_018914.2|
	TESTI2 cDNA clone:	Dihydrofolate reductase isoform1 and 3:
	Seq ID: dbj|DB053698.1|	Seq ID: ref|NC_018914.2|
DQ585088:		
piR-52200	EEF1A: Seq ID: ref|XM_011535514.1|	SLC1A5: sequence ID: ref|XM_005259167.3|
	Phosphatidylinositol 4,5-bisphosphate 3-kinase	ZDHHC17: sequence ID:ef|XM_005268749.3|
	Seq ID: ref|NC_018914.2|	myc box-dependent-interacting protein 1
		isoform 7: Seq ID: ref|NC_018913.2|
DQ597061		
piR-35127	104216 bp at 5’ side: promotilin isoform 1	ubiquitin-conjugating enzyme (UBE2V2):XM_011517583.1
	117238 bp at 3′ side: metabotropic glutamate receptor 4 isoform 2	
	Seq ID: ref|NC_018917.2|	
DQ578433:		
piR-46545	Lipopolysaccharide-responsive and beige-like anchor protein gene:	NEIL2: Seq ID: ref|XM_005272383.1|
		UPB1: Seq ID: ef|XM_011530225.1|
	Seq ID: ref|NC_018915.2|	ITGB5: Seq ID: ref|NM_002213.4|
DQ583373:		
piR-50485	ZHX3: Seq ID: ref|XM_011528720.1|	PHACTR1: Seq ID: ref|XM_006715021.2|
		TRHR: Seq ID: ref|XM_011517263.1|

A 100% sense match (plus/plus) is considered a potential source of the pRNA. For piRNA targets, a 13 consecutive nucleotide (plus/minus) or > match is considered a potential piRNA target gene.

### Over-expression and knock-down of RASSF1C target piRNAs

**Figure 5:**
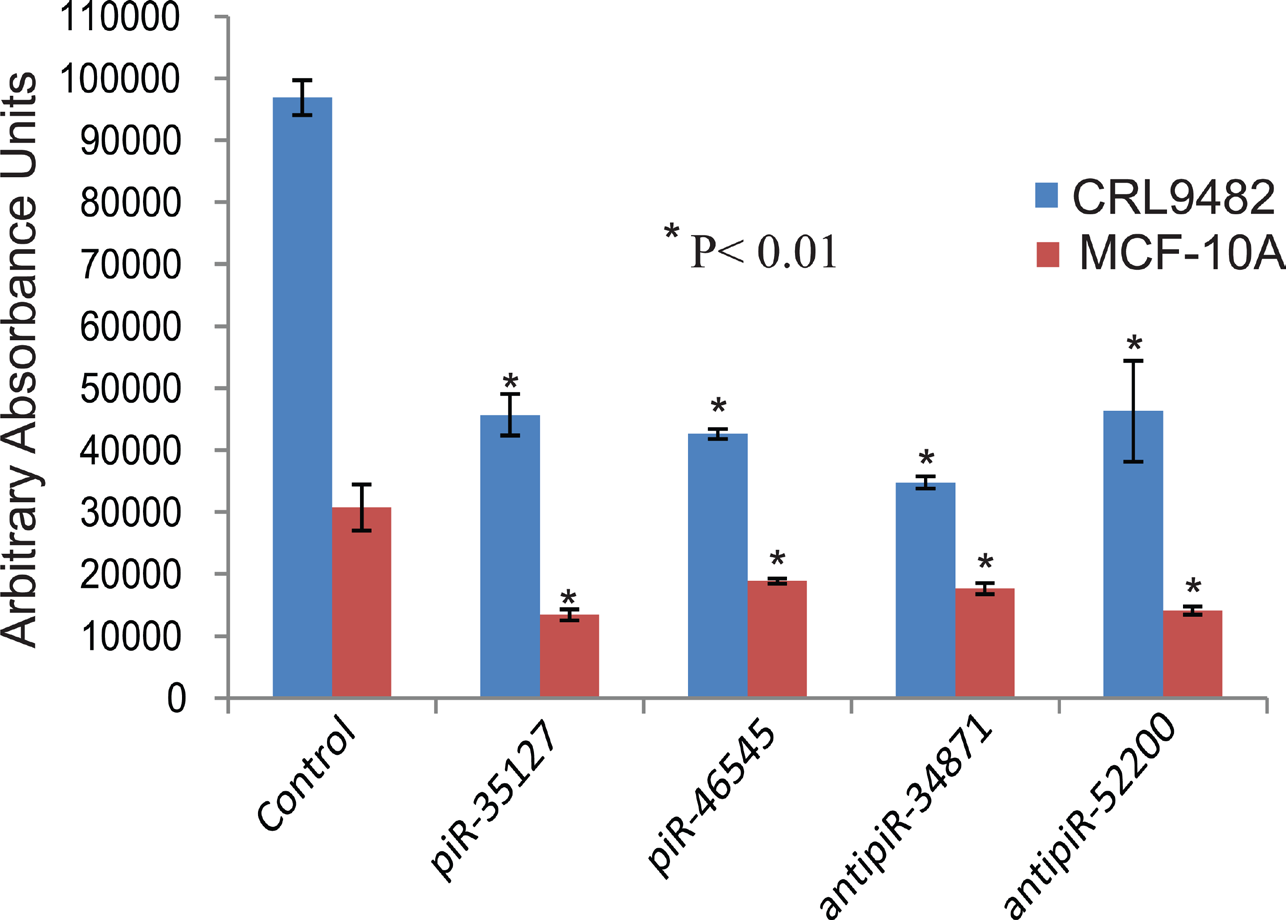
Down-regulation of piR-34871 and piR-52200 and over-expression of piR-35127 and piR-46545 in normal lung epithelial cells (CRL9482) and mammary epithelial cells (MCF-10A) Treatment of CRL9482 and MCF-10A cells with 500 nM of anti-piR-34871 or anti-piR-52200 RNA mimics or with sense piR-35127 or piR-46545 RNA mimics significantly decreased cell proliferation as determined by Alamar Blue assay 48 h post-transfection. All experiments were done at least 3 independent times with *n* = 4 wells per treatment. The (*) indicates statistical significance compared to controls (scrambled piRNA), with a *P <* 0.01.

**Figure 6:**
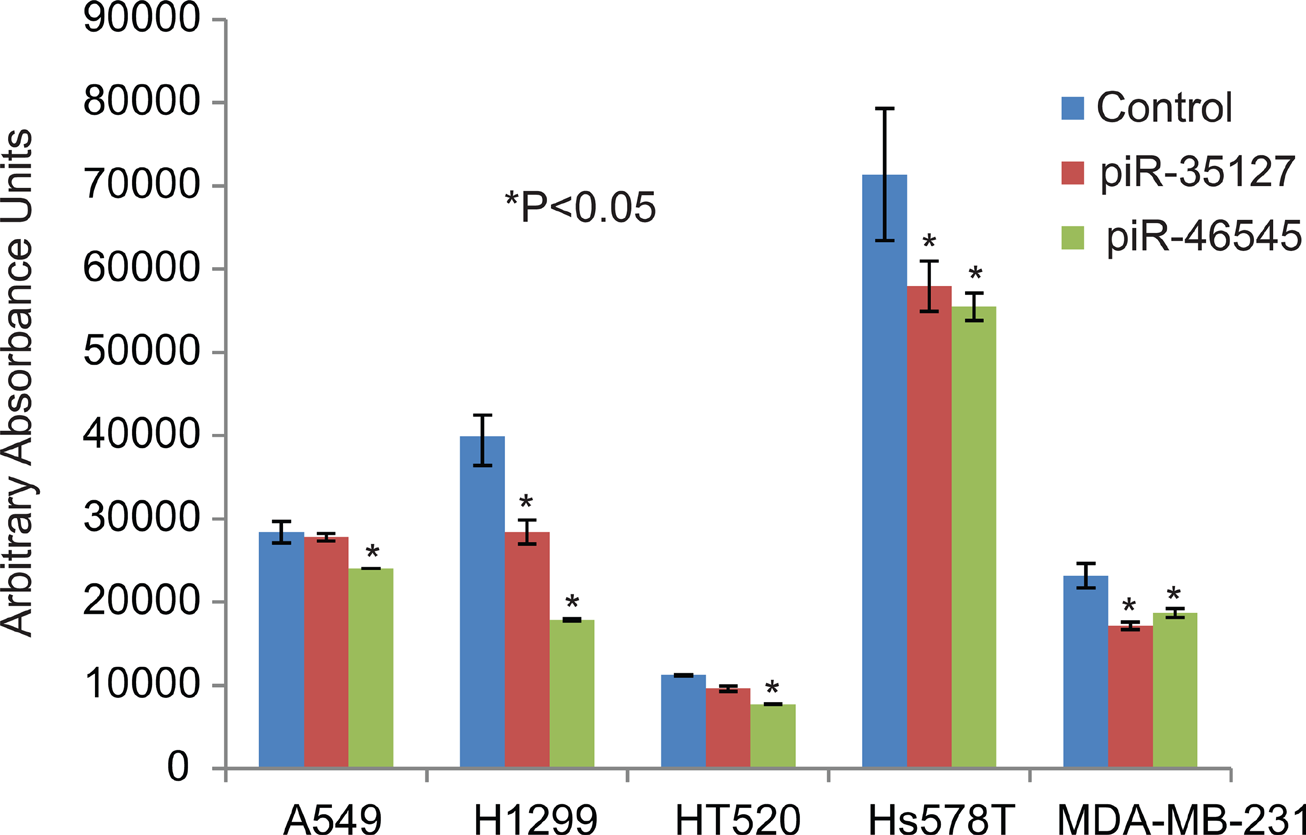
Over-expression of piR-35127 and piR-46545 in lung cancer cells Treatment of lung cancer cell lines (A549, H2199, and HT520) and breast cancer cell lines (Hs578T and MDA-MB-231) with 500 nM of piR-35127 and piR-46545 mimic RNA oligos decreased cell proliferation of lung cancer cells as determined by the Alamar Blue assay. All experiments were done at least 3 independent times with *n* = 4 wells per treatment. The (*) indicates statistical significance compared to controls (scrambled piRNA), with a *P <* 0.05.

**Figure 7:**
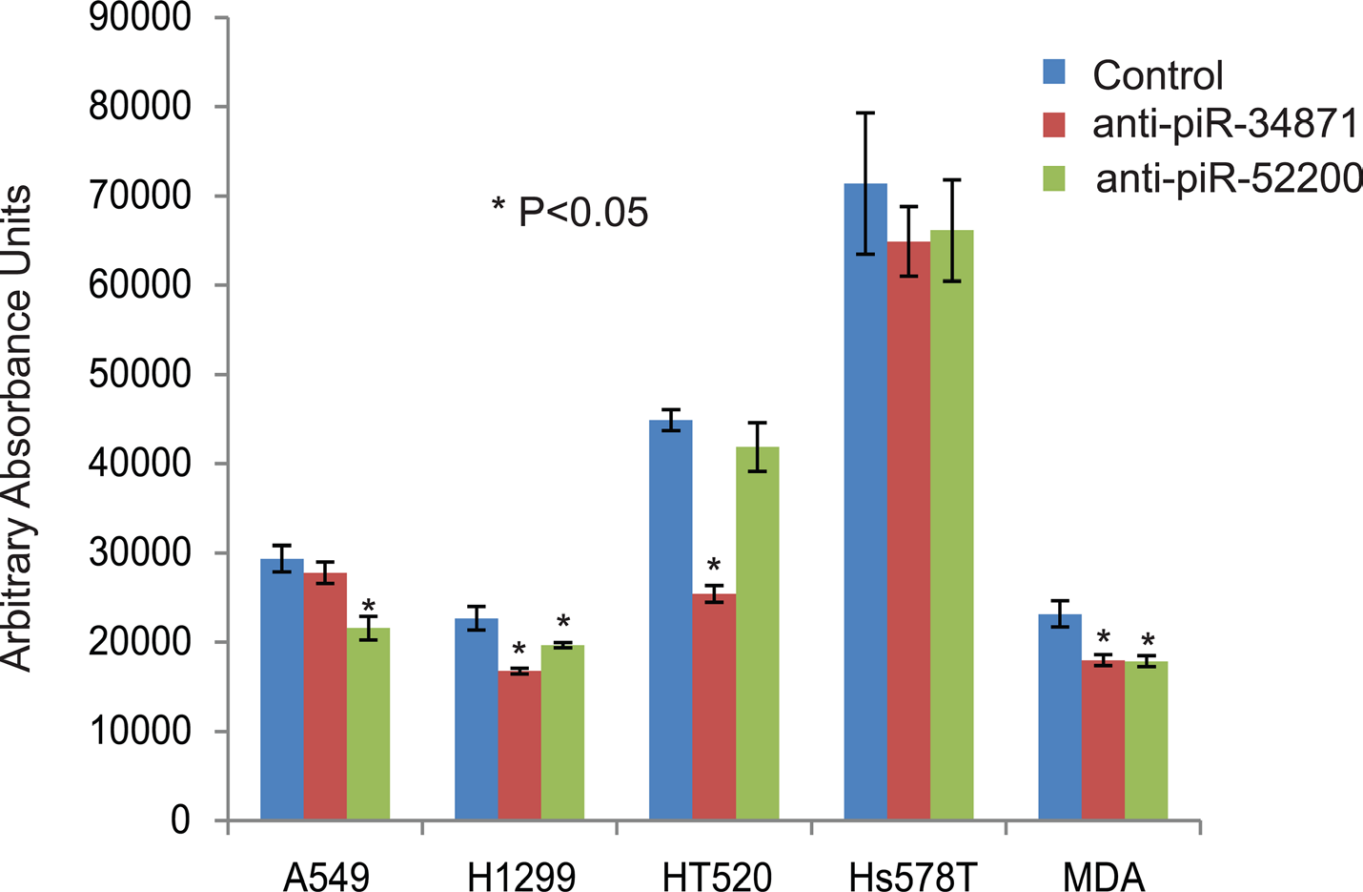
Down-regulation of piR-34871 and piR-52200 in lung and breast cancer cells Treatment of lung cancer cell lines (A549, H2199, and HT520) and breast cancer cell lines (Hs578T and MDA-MB-231) with 500 nM of anti-piR-34871 and anti-piR-52200 mimic RNA oligos. Anti-piR-34871 significantly decreased cell proliferation of H1299, H520, and MDA-MB-231 but not A549 or Hs578T cells. Anti-piR-52200 significantly decreased cell proliferation of A549, H1299, and MDA-MB-231 cells but not HT520 or Hs578T cells as determined by the Alamar Blue assay. All experiments were done at least 3 independent times with *n* = 4 wells per treatment. The (*) indicates statistical significance compared to controls (scrambled piRNA), with a *P <* 0.05.

To determine the function of the RASSF1C down-regulated piRNAs, we over-expressed piR-35127 and piR-46545 in normal lung and breast epithelial cell lines as well as lung and breast cancer cell lines. Over-expression of piR-35127 and piR-46545 in normal lung and breast epithelial cells (Figure 5) and in a lung cancer cell line (H1299) and breast cancer cells lines (Hs578T and MDA-MB-231, Figure 6) resulted in a significant reduction in cell proliferation. Over-expression of piR-35127 did not affect proliferation of the lung cancer cell lines A549 and HT520 (Figure 6). We also evaluated whether the up-regulation of piRNAs by RASSF1C affects cell proliferation. As such, piR-34871 and piR-52200 were knocked down in normal lung and breast epithelial cells (Figure 5) and lung and breast cancer cells (Figure 7). Knockdown of piR-34871 and piR-52200 expression significantly decreased cell proliferation of normal lung and breast epithelial cells. Knock-down of piR-34871 significantly reduced cell proliferation of H1299, HT520, and MDA-MB-231 cells, but did not have a significant effect on A549 and Hs578T cell proliferation. On the other hand, knock-down of piR-52200 resulted in a significant reduction in cell proliferation of A549, H1299, and MDA-MB-231, but it did not have a significant effect on HT520 and Hs578T cell proliferation.

### Impact of piRNAs on cell colony formation

**Figure 8:**
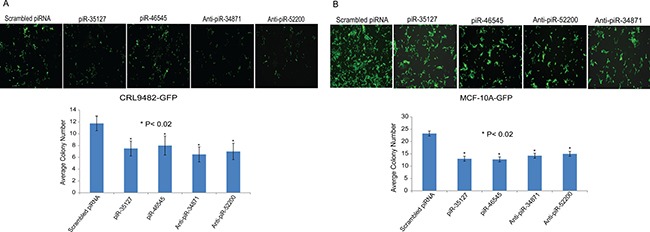
Impact of piR-35127, piR-46545, anti-piR-34871, and anti-piR-52200 on colony formation Normal lung epithelial cells (CRL9482, panel A) and mammary epithelial cells (MCF-10A, panel B) stably over-expressing green fluorescent protein (GFP) were plated in 96-well plates and were transfected with 500 nM of scrambled piRNA (control), piR-35127, piR-46545, anti-piR-34871, or anti-piR-52200. Cells were imaged and photographed 5–7 days post-transfection using a fluorescent microscope. Cell colonies in four microscopic fields per well were counted and the average cell colony number count of control and experimental was plotted. Cells transfected with the four piRNAs formed significantly fewer colonies compared to cells transfected with the scrambled piRNA. The data suggests that over-expression of piR-35127 and piR-46545 and knock-down of piR-34871 and piR-52200 attenuates cell proliferation. All experiments were done at least 3 independent times with *n* = 4 wells per treatment. The (*) indicates statistical significance compared to controls, with a *P <* 0.02.

We also measured the impact of over-expressing piR-35127 and piR-46545 and knocking down piR-34871 and piR-52200 on normal lung and breast epithelial cell colony formation. Normal lung and breast epithelial cells stably over-expressing green fluorescent protein (GFP) were transfected with scrambled piRNA (control), piR-35127, piR-46545, anti-piR-34871, or anti-piR-52200. Cells were imaged and photographed 5–7 days post-transfection using a fluorescent microscope. Both over-expression of piR-35127 and piR-46545 and knock-down of piR-34871 and piR-52200 resulted in a significant reduction of lung and breast cell colony formation (Figure 8). The data further supports our hypothesis that these RASSF1C piRNA target genes modulate cell proliferation of both lung and breast.

### Effect of piRNAs on Caspase 3/7 activation

**Figure 9:**
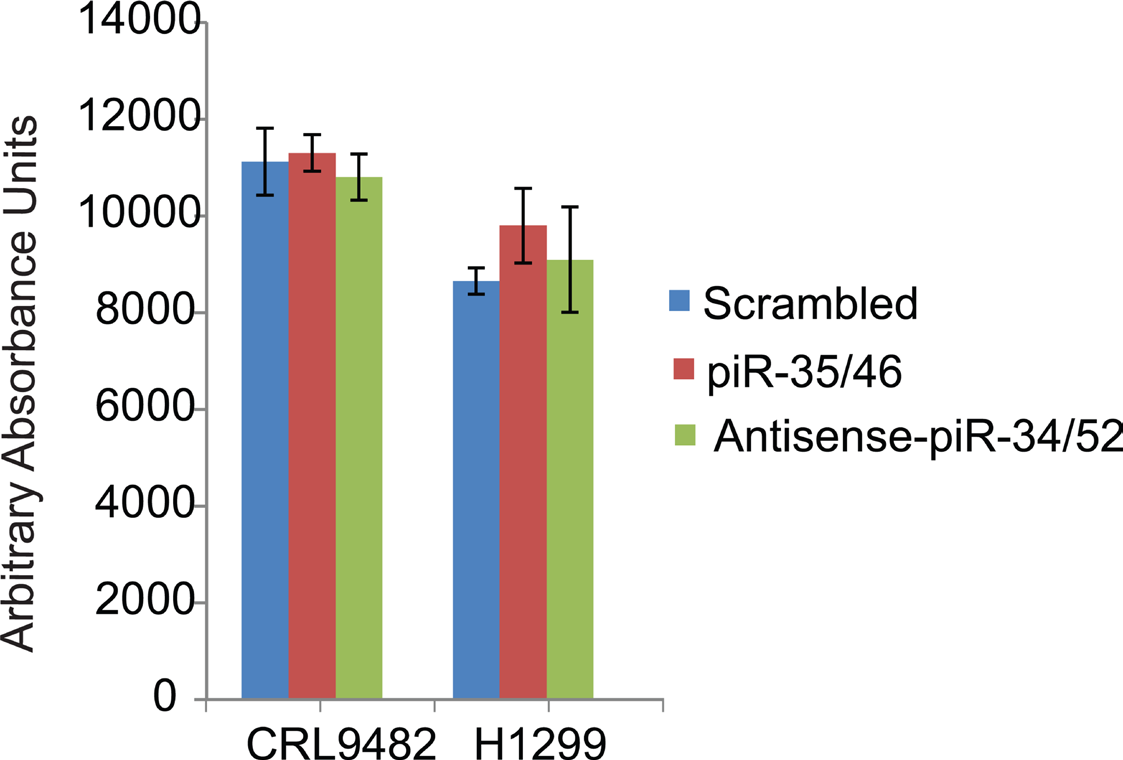
Effect of piRNAs on Caspase 3/7 activation Caspase 3/7 activation was assessed in normal lung epithelial cells (CRL9482) and in lung cancer cells (H1299). Over-expression of piR-35127 and piR-46545 or down-regulation of piR-34871 and piR-52200 did not have an impact on caspase 3/7 activation. All experiments were done at least 3 independent times with *n* = 4 wells per treatment. The values obtained were not statistically significant as indicated by the error bars which represent the mean ± SEM.

We also assessed cells treated with the piRNAs for caspase 3/7 activity and we found that the caspase 3/7 activation was not impacted by co-expression of piR-35127 and piR-46545 or by co-knocking down piRNA-34871 and piR-52200. This suggests that these piRNAs are not modulators of apoptosis (Figure 9). Taken together, our data suggest that down-regulation of piR-35127 and piR-46545 and up-regulation of piR-34871 and piR-52200 may promote cell proliferation of lung cancer cells and that this could be one potential mechanism through which RASSF1C contributes to oncogenesis and progression of lung cancer. The current findings further support our working hypothesis that PIWIL1 and piRNA are downstream targets of RASSF1C and suggest a novel mechanism in lung cancer oncogenesis and progression.

### RASSF1C over-expression attenuates AMPK-α phosphorylation

**Figure 10:**
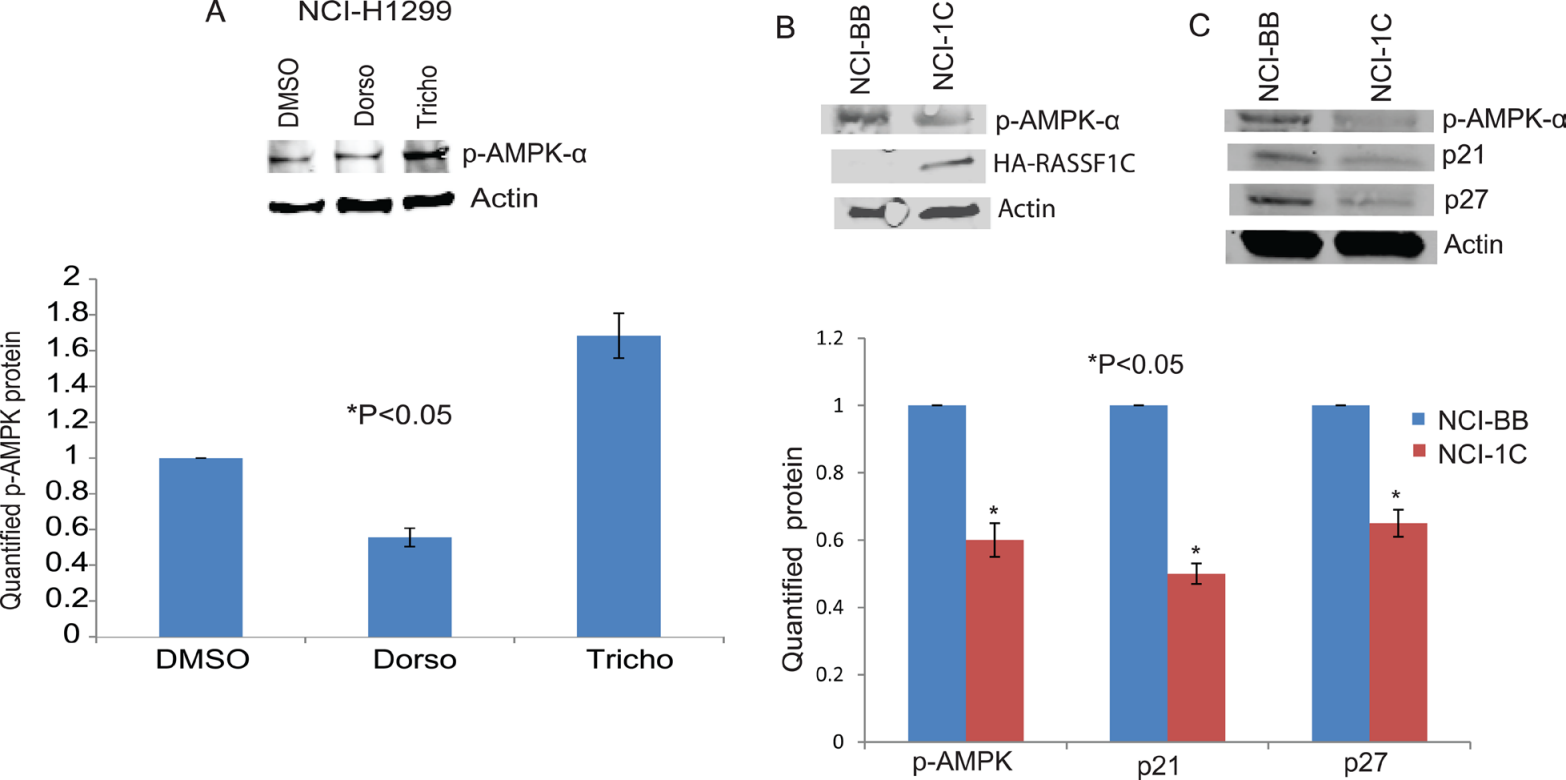
AMPK phosphorylation (**A**) Western blot analysis of p-AMPK in NCI-H1299 cells treated with DMSO, Dorsomorphin (Dorso) or Trichostatin A (Tricho). Dorsomorphin reduces and Trichostatin increases pAMPK levels. Quantified levels of p-AMPK were determined as an average signal (DMSO vs Dorso and Tricho) from at least 3 independent blots, with a *P <* 0.05. (**B**) Western blot analysis of p-AMPK in NCI-H1299 cells over-expressing RASSF1C (NCI-1C) showed reduced pAMPK levels compared to vector backbone control (NCI-BB). (**C**) Western blot analysis of p21 and p27 in NCI-H1299 cells over-expressing RASSF1C (NCI-1C) showed reduced p21 and p27 levels compared to vector backbone control (NCI-BB). Quantified levels of p-AMPK, p21, and p27 were determined as an average signal (NCI-BB vs NCI-1C) from at least 3 independent blots, with a *P <* 0.05.

In previously published work, we have identified that Dorsomorphin (AMPK inhibitor) up-regulates and Trichostatin A (HDAC inhibitor and AMPK activator) down-regulates RASSF1C and PIWIL1 mRNA levels in lung cancer cells [17]. Therefore, we assessed the impact of over-expressing RASSF1C on AMPK activation (phosphorylation) and found that over-expression of RASSF1C leads to a reduction in AMPK-α phosphorylation, which is a similar effect to that observed when treating lung cancer cells with Dorsomorphin (Figure 10). We also assessed the expression of the kinase inhibitor proteins, p21 and p27, which are downstream effectors of the ATM- AMPK-p53/p21cip1 pathway. We found that over-expression RASSF1C appears to also decrease the expression of p21 and p27 (Figure 10).

**Figure 11:**
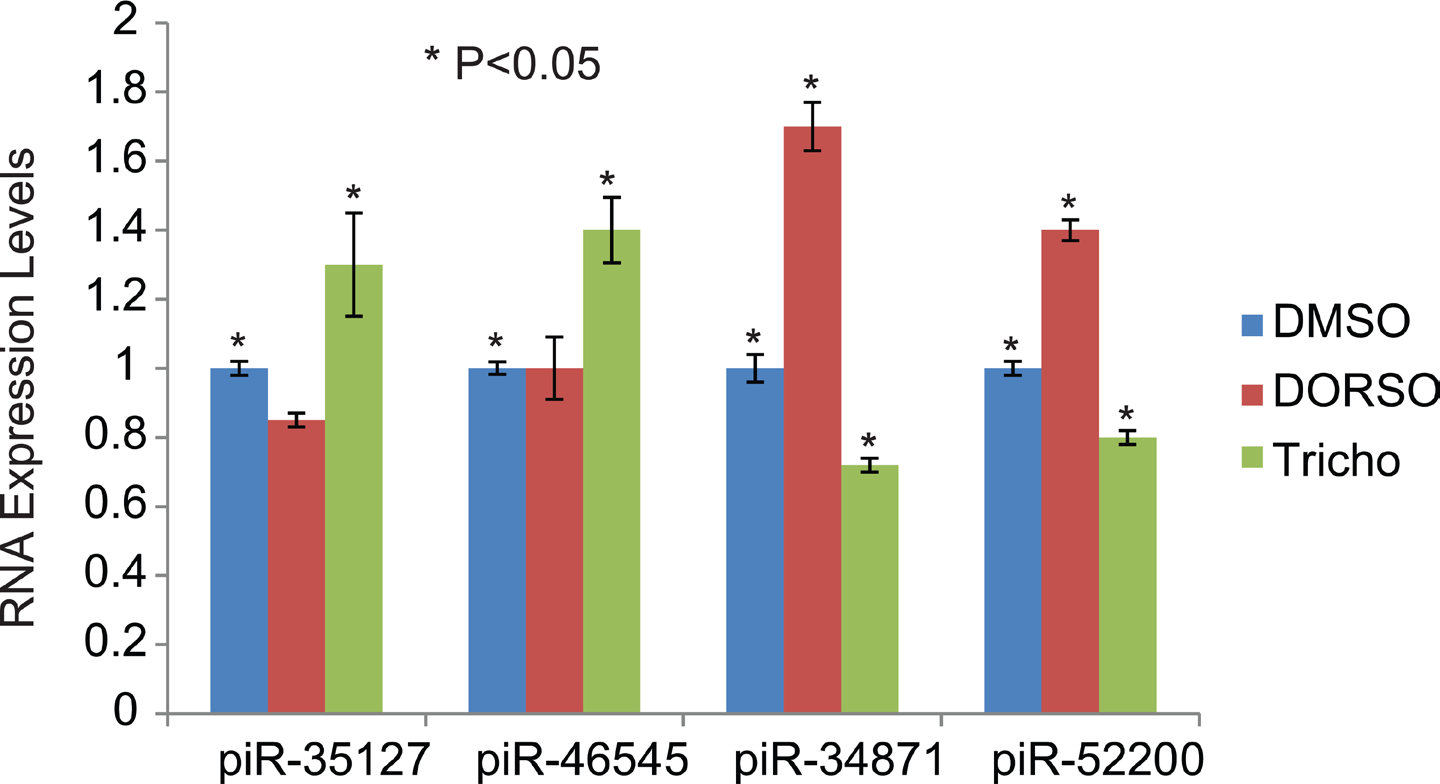
Modulation of piRNA expression by Dorsomorphin and Trichostatin H1299 lung cancer cells were treated with Dorsomorphin (Dorso) or Trichostatin (Tricho) and the expression of specific piRNAs was assessed. Dorsomorphin treatment increased the expression of piR-34871 and piR-52200 but did not have a significant effect on piR-35127 and piR-46545. Trichostatin A increased the expression of piR-35127 and piR-46545 and decreased the expression levels of piR-34871 and piR-52200. The RT-PCR of control and experimental reactions were run in triplicates in multiple independent runs and the 2^-ΔΔ^ method was used to perform statistical analysis (32). The (*) indicates statistical significance compared to controls (DMSO), with a *P <* 0.05.

We have also assessed the effect of Dorsomorphin and Trichostatin A on the expression of piRNA that are down-regulated by RASSF1C (piR-35127, piR-46545) and piRNAs that are up-regulated by RASSF1C (piR-34871 and piR-52200). We found that Dorsomorphin treatment increased the expression of piR-34871 and piR-52200 while Trichostatin A increased the expression of piR-35127 and piR-46545 and decreased the expression levels of piR-34871 and piR-52200 (Figure 11). Our findings suggest that RASSF1C may modulate the expression of these piRNAs, in part, through modulation of the AMPK pathway.

## DISCUSSION

RASSF1C is an emerging oncoprotein with potential functions involved in regulating cell proliferation, apoptosis, migration, and cell cycle progression [14–17]. In previously published work, we have reported that RASSF1C promotes lung and breast cancer cell growth, in part, through the up- and down-regulation of important target genes involved in lung and breast cancer growth and progression [14, 18]. Recently published work shows that RASSF1C modulates SRC activity to promote breast tumorigenesis both *in vitro* and *in vivo* [27], which is consistent with and lends support to our previously published work [16, 18]. One of the RASSF1C target genes in lung cancer cells that we have been studying in our laboratory is the stem cell renewal gene, *piwil1*. The up-regulation of the *piwil1* gene by RASSF1C suggests that RASSF1C may play a role in promoting lung cancer stem cell development and progression [14, 17]. Since PIWIL1 interacts with small noncoding RNAs known as PIWI-interacting RNAs (piRNAs) to regulate the expression of target genes, we wanted to determine if RASSF1C also modulates the expression of piRNAs as well. In this regard, we performed a global piRNA array screen using NSCLC H1299 cells over-expressing RASSF1C to determine if RASSF1C also modulates the expression of specific piRNAs. The piRNA microarray screen data shows that over-expression of RASSF1C appears to influence the expression of over 500 piRNAs in lung cancer cells, some of which are listed in Table 1. To begin the functional characterization of some of the identified piRNAs, we first validated the expression of the two most up-regulated piRNAs (piR-34871 and piR-52200) and the two most down-regulated pi-RNAs (piR-35127 and piR-46545) in lung cancer cells that either over-express RASSF1C, RASSF1A, and in cells with knocked-down RASSF1C expression by RT-PCR (Figure 3). We confirmed that over-expression of RASSF1C up-regulates piR-34871 and piR-52200 and down-regulates piR-35127 and piR-46545 while the opposite is true in cells with knocked-down RASSF1C expression with respect to piR-35127 and piR-46545. Over-expression of RASSF1A down-regulated piR-52200 but did not impact the expression of piR-34871, piR-35127, or piR-46545.

Furthermore, we assessed the expression profile of these four piRNAs, along with RASSF1A and RASSF1C expression, in human lung tumor tissue. The expression prolife analysis showed that there was some correlation between RASSF1C expression, up-regulated in 50% of the tumor tissues, and its piRNA target genes in some tumor tissues but not in all (Figure 4). The data show that the expression of piR-34871 and piR-52200 were up-regulated in about 50–58% and down-regulated in 40% of tumor samples tested (*n* = 12), while the expression of piR-35127 and piR-46545 were down-regulated in approximately 50% of tumors. Interestingly, there was a remarkable inverse correlation between the expression of RASSF1C and piR-35127 in 10 out of the 12 tumor tissues tested in this study. This suggests that piR-35127 may in fact be an important target gene for RASSF1C. In contrast, the expression of RASSF1A did not seem to correlate with the expression of any of the four piRNAs being profiled (Figure 4A). We also determined the ratio of RASSF1C to RASSF1A expression and found that RASSF1C/RASSF1A ratio was > 1 in 58% (Figure 4B) of tumor tissues tested in this study. Obviously, further expression analysis of piRNAs using a larger cohort is needed to definitively determine and validate RASSF1C modulation of the piRNA target genes identified in this study; and to further investigate the impact of the RASSF1C/RASSF1A expression ratio on piRNA gene expression.

To learn about the genomic source(s) of the piRNAs, we carried out sequence alignment analysis using mature piRNA sequences to search the human genome and transcriptome sequence data banks. The sequence alignment analysis identified potential candidate genes as sources for the piRNA transcripts. For example, we found that piR-34871 could be derived from both a mitochondrial gene as well as nuclear genes. PiR-52200, piR-35127, and piR-46545 appear to be solely derived from nuclear genes. We have also identified potential mRNA targets for these piRNAs that show at least 13 consecutive complementary nucleotides in the 3′ untranslated region (Table 2). We are in the process of validating and confirming potential mRNA targets for these novel piRNAs.

Because we found that piR-35127 and piR-46545 were down-regulated in lung cancer cells over-expressing RASSF1C and were also down-regulated in 50% of lung tumor tissues examined, we over-expressed piR-35127 and piR-46545 in lung and breast cancer cell lines and in normal lung and breast epithelial cells and assessed their impact on cell proliferation, colony formation, and apoptosis. Over-expression of piR-35127 and piR-46545 resulted in a significant reduction of normal lung and breast epithelial cells (Figure 5). Further, over-expression of piR-35127 reduced cell proliferation of H1299, Hs578T, and MDA-MB-231 but had no effect on A549 and HT520 cells. Over-expression of piR-46545 reduced A549, H1299, HT520, Hs578T, and MDA-MB-231 (Figure 6). We also assessed the impact of knocking down the expression of piR-34871 and piR-52200. Knocking down of piR-34871 and piR-52200 expression resulted in reduced cell proliferation of normal lung and breast epithelial cells cancer cells (Figure 5). Knocking down piR-34871 reduced cell proliferation of H1299, HT520, and MDA-MB231 but had no effect on A549 and Hs578T cells. Knocking down piR-52200 reduced cell proliferation of A549, H1299, and MDA-MB-231, but had no effect on HT520 and Hs578T cells. The observation that some cancer cell lines, but not the normal cells, used in this study appear to be impacted differently by the four piRNAs being tested is interesting and may underscore the genetic instabilities/defects that these cancer cell lines harbor. None the less, we think the data is useful in identifying the most suitable cell model to study the function of a specific piRNA.

In addition to performing cell proliferation assays, we also assessed the impact of over-expressing piR-35127 and piR-46545 and knocking down piR-34871 and piR-52200 on normal lung and breast epithelial cell colony formation. We found that over-expression of piR-35127 and piR-46545 and knock-down of piR-34871 and piR-52200 resulted in a significant reduction of lung and breast cell colony formation (Figure 8, *P <* 0.02), which is consistent with the cell proliferation data obtained by the Alamar Blue assay (Figure 5). This data further supports our hypothesis that these novels piRNAs modulate cell proliferation.

Furthermore, we investigated the impact of these piRNAs on apoptosis by measuring caspase 3/7 activation in normal and lung cancer cells. We found that neither co-over-expression of piR-35127 and piR-46545 nor co-knock-down of piR-34871 and piR-52200 resulted in any significant increase of caspase 3/7 activation (Figure 9). This suggests that these piRNAs may not be involved in modulating cell apoptosis and that they may be involved in cell cycle regulation which is consistent with our published work showing that RASSF1C promotes cell cycle progression of lung cancer cells [13]. Further, expression modulation of certain piRNAs may promote lung epithelial-mesenchymal transition (EMT), which is a prerequisite for epithelial cell transformation. Indeed, PIWIL1 protein has been reported to play a role in modulating EMT and the promotion of endometrial cancer stem cells [28]. Thus, it is also of interest to assess the impact of the RASSF1C-PIWIL1-piRNA axis in promoting lung cancer stem cell development.

In previously published work, we reported that Dorsomorphin (AMPK inhibitor) and KU-60019 (ATM kinase inhibitor) up-regulate and Trichostatin A (HDAC inhibitor and AMPK activator) down-regulates RASSF1C mRNA levels in lung cancer cells [17]. In this study we assessed the impact of Dorsomorphin and Trichostatin A on piRNA gene expression in lung cancer cells. We found that lung cancer cells treated with Dorsomorphin showed increased levels of piR-34871 and piR-52200 (both of which are up-regulated by RASSF1C), while cells treated with Trichostatin A showed decreased expression of piR-34871 and piR-52200 and increased expression of piR-35127 and piR-46545 (Figure 11). We also found that over-expression of RASSF1C, like Dorsomorphin, resulted in a reduction of AMPK phosphorylation as well as p21 and p27 protein levels (Figure 10). Thus, our findings suggest that attenuation of the AMPK pathway by RASSF1C may be a potential mechanism through which RASSF1C exerts its actions in lung cells. AMPK phosphorylation is an important component of the ATM-AMPK-p53-p21^cip^ pathway. Activation of the of the ATM-AMPK-p53-p21^cip^ pathway results in the inhibition of pro-survival growth pathways such as the Akt-mTOR-4EBP1 pathway; and it also results in inhibition of lung cancer cell proliferation and induction of cell cycle arrest [29, 30]. Reduced phosphorylation of AMPK has been reported in gastric and lung cancers. Restoration of AMPK phosphorylation in lung cancer cells leads to down-regulation of the transcription regulator Bmi-1, which has been shown to promote EMT and its expression is associated with cell progression [30]. Recent work also shows that AMPK activation sensitizes EGFR wild type H1299 cells and tumors to erlotinib treatment, suggesting a role of AMPK activation in modulating EGFR signaling and drug sensitivity in lung cancer cells [31]. We should note that in previously published work, we identified EGFR as one of the target genes that is up-regulated in H1299 over-expressing RASSF1C [14]. Thus, RASSF1C-induced reduction of AMPK phosphorylation as well as p21 and p27 protein levels may potentially attenuate the ATM-AMPK-p53-p21^cip^ pathway and may also enhance EGFR signaling leading to blocking cell cycle arrest and promoting cell proliferation.

It is tempting to speculate that the modulation of the PIWIL1-piRNA gene axis by RASSF1C may play a role in promoting EMT, which is a prerequisite for epithelial cell transformation. It is also of interest to determine if any of these novel piRNAs identified actually load on PIWIL1 protein and modulate their target genes in lung stem cells. As mentioned above, we have previously reported that RASSF1C modulation of the *piwil1* gene may elevate beta-catenin expression leading to lung cancer growth and progression. Identifying specific piRNAs that are modulated by RASSF1C will enhance our understanding of how the RASSF1C-PIWIL1-piRNA axis impacts lung cancer cell proliferation, apoptosis, invasion, and migration in ways that have not been previously reported in literature.

## MATERIALS AND METHODS

### Cell culture

The human lung cancer cell line NCI-H1299 stably over-expressing HA-RASSF1C, GFP, or vector back bone was grown in RPMI-1640 medium supplemented with 10% calf bovine serum. The human lung epithelial cell line CRL9482 was grown in BEMB media as previously described [14, 17]. The A549 lung cancer cell line was grown in F-K12 media supplemented with 10% FBS as previously described [14, 17]. The HT520 lung squamous cancer cell line was grown in RPMI-1640 medium supplemented with 10% fetal bovine serum. MCF-10A normal mammary epithelial cell line was grown in MEMB, the breast cancer cell line MDA-MB-231 was grown in DMEM with 10% calf bovine serum, the breast cancer cell line Hs578T was grown RPMI-1640 medium supplemented with 10% calf bovine serum.

### Total RNA isolation

Total RNA was isolated from H1299 cells stably over-expressing RASSF1C or vector back bone as previously described [14]. The isolated RNA was submitted to Arraystar (Rockville, MD) for the piRNA microarray screen and data analysis.

### Human piRNA array

The Arraystar HG19 piRNA array, which is designed for profiling 23000 human piRNAs (ArrayStar, Rockville, MD), was used for this study.

### RNA labeling and hybridization

Sample labeling was performed using an RNA ligase method. Briefly, 1 microgram of each sample was 3′-end-labeled with Cy3 fluorescent label using Quick Amp Labeling Kit (Agilent Technologies, Santa Clara, CA). The labeling reaction was incubated for 1 h at 16°C, and terminated by incubation for 15 min at 65°C. After stopping the labeling procedure, the Cy3-labeled samples were hybridized to the Arraystar Human piRNA Array. Hybridization was performed at 65°C for 17 h in Agilent's SureHyb Hybridization Chambers. Slides were washed in an ozone-free environment and were fixed and scanned using the Agilent DNA Microarray Scanner (part number G2505C).

### Data collection and normalization

After washing, slides were scanned with the Agilent DNA Microarray Scanner. Data was extracted using Agilent Feature Extraction software (version 11.0.1.1, Agilent Technologies). Quantile normalization and subsequent data processing were performed using the GeneSpring GX v11.5.1 software package (Agilent Technologies). After quantile normalization of the raw data, piRNAs that had at least 3 out of 6 samples with flags in Present or Marginal (“All Targets Value”) were chosen for differentially expressed piRNAs screening.

### Lung tumor and matched normal tissues

Tumor and matched normal tissues were obtained from the Western division of the Cooperative Human Tissue Bank (Vanderbilt University, TN). Tumor and normal tissues were ground to a powder using a mortar and pestle, and the tissue powder was used to isolate total RNA using PureLinkTM RNA Mini Kit [Invitrogen, Carlsbad, CA] as previously described [15].

### RT-PCR analysis

Total RNA from human lung cancer cells, lung tumor and matched normal tissues was isolated and reverse transcriptase (RT)-PCR was performed using RASSF1C gene-specific primers as previously described (14). PCR was carried out using HotStart and SYBR Green master mixes (Qiagen, Valencia, CA). For piRNA expression analysis, reverse transcriptase (RT) was performed using Quantimir RT kit (System Biosciences, Mountain View, CA). PCR was performed using KAPA SYBR^R^ FAST qPCR Kit (KAPA Biosystems, Boston, MA). The first 21 nucleotides of each piRNA sequence were used as a forward primer, along with a universal reverse primer included in the Quantimir RT kit. The piRNA primers were: piR-34871: GAGTAGAGTGCTTAGTTGAACAGA, piR-46545: TCTTTCACGATGGTGCAATTCAA, piR-50485: TGCACAGAGACACACCCACACTA, piR-35127: GCA CTCAGAAACACACATGCTCA, and piR-52200: TGC CTGGGTCTTGGATAAACTGA. The RT-PCR reactions were carried out in triplicates and the fold change was calculated using the 2^-ΔΔCT^ method [32].

### Cell transfection

Transfection of cells with piRNA oligos at 500 nM final concentration was carried out using lipofectamine (Invitrogen, Carlsbad, CA) or Mirus (Mirus Bio LLC, Madison, WI). Cells were plated at 2500–5000 cells per well (*n* = 8) and cells were transfected the next day and were incubated for 48 h before performing the Alamar Blue assay as previously described [13]. The sequence of the piRNA oligos used was as follows:

Scrambled piRNA oligo: /5Phos/GrUGCrUAG GrUACGrUCArUCmU

Antisense piR-34871 RNA oligo: /5Phos/GGCCCrU GrUrUCAACrUAAGCACrUCrUACrUmC

Antisense piR-52200 RNA oligo: /5Phos/GCrUrUr UCAGrUrUrUArUCCAAGACCCAGGCmA

Sense piR-35127 RNA oligo: /5Phos/GCACrU CAGAAACACACArUGCrUCAGCCmC

Sense piR-46545 RNA oligo: /5Phos/rU CrUrUrUCACGArUGGrUG CAArUrUCAACAGGAmU

### Cell proliferation assay

Cell proliferation was measured by the Alamar Blue assay as previously described [18]. Data are presented as mean values ± SEM and analyzed with Student's *t-test*. Values ≤ 0.05 were considered significant.

### Colony formation assay

CRL9482 and MCF-10A cell lines stably over-expressing GFP were treated with sense piRNAs (piR-35127, piR-46545) or antisense piRNAs (piR-34871 and piR-52200) at a concentration of 500 nM. Cells were plated at 2000 cells per well (*n* = 4) and cells were transfected the next day and incubated for 5–7 days. Cells were imaged 5–7 days post-transfection using fluorescence microscopy with a wide field imaging system (Leica microsystems Inc, Buffalo Grove, IL). Cell colonies in four microscopic fields per well were counted and the average cell colony number of control and experimental was plotted.

### Caspase 3/7 activity assay

Cells transfected with scrambled piRNA (control) and piRNAs (piR-35127/piR-46545 and antipiR-34871/anti-piR-52200) were assayed for caspase 3/7 activity using the Apo3/7 caspase activity assay 48 h post-transfection (Promega, Madison, WI) as previously described [15].

### Dorsomorphin and Trichostatin A treatment

H1299 lung cancer cells were treated with Dorsomorphin (10 μM) or Trichostatin A (10 μM) in serum free media for 24 h before cells were collected for RNA isolation. Control cells were treated with Dimethyl sulfoxide (DMSO). Dorsomorphin was obtained from Phoenix Pharmaceutical, Inc. (Burlingame, CA) and Trichostatin A was obtained from Reagents Direct (Encinitas, CA). Total RNA was isolated as previously described [14] and microRNA was isolated using PurLink microRNA isolation kit (Invitrogen) from treated cells. The expression analysis of piRNAs was carried out as described above. The experiments were repeated at least 3 times.

### Western blot analysis

Western blot analysis of experimental and control cell lysates was carried out using the Odyssey^®^ Infrared System (LI-COR Biosciences, Lincoln, NE) and polyclonal anti-phospho-AMPK-α (Thr 172) antibody (Cat # 07-681SP), polyclonal Anti-p21 antibody (Cat # 0506550), and polyclonal Anti-Kip1 (p27) (Cat # 060445) were purchased from Millipore (Millipore Corporation, Iselin, NJ ). Polyclonal beta actin antibody (Cat # sc-1615) and polyclonal RASSF1C antibody (sc-18724) were purchased from Santa Cruz Biotechnology, Inc (Santa Cruz, CA), and fluorescently-labeled secondary antibodies IRDye^®^ 680 and 780 RD Infrared Dye were purchased from LI-COR (LI-COR Biosciences, Lincoln, NE). The experiments were repeated at least 3 times. Protein levels were normalized to actin levels (the loading control).

### Statistical analysis

The *t-test* was used to calculate the significance of the data.

## CONCLUSIONS

In this study we report on new and novel findings in regard to the identification of several RASSF1C piRNA target genes in the lung cancer cell line H1299. The findings reported in this article present a new dimension to the role of RASSF1 gene in cancer. We have begun studies of some of these piRNAs identified. We have validated the expression of four piRNAs by RT-PCR in cancer cells and have assessed their expression in lung tumor and matched normal tissues. We found that the expression of piR-35127 was down-regulated in 10/12 tumor tissues and its expression is inversely correlated with RASSF1C. Over-expression of piR-35127 and piR-46545 and silencing of piR-34871 and piR-5200 significantly reduced proliferation of normal lung and breast epithelial cells, suggesting a role in lung and breast cell growth. We also found that RASSF1C may promote its activities, in part, through attenuation of the ATM-AMPK-p53-p21^cip^ pathway. Linking a RASSF1C-PIWIL1-piRNA axis to lung epithelial or stem cell transformation and progression is a novel and new concept that upon further investigation could lead to discovery of new diagnostic and therapeutic targets for lung cancer.
